# Effect of nurse-led intervention on knowledge and preventive behavior of diabetic pregnant women regarding COVID-19 associated mucromycosis infection in mid-delta region of Egypt

**DOI:** 10.1186/s12912-023-01320-x

**Published:** 2023-05-22

**Authors:** Marwa A. Shahin, Mira M. Abu-Elenin, Hanan E. Nada

**Affiliations:** 1 Department of Maternal and Newborn Health Nursing, Faculty of Nursing, Menoufia University, Shebin El-Kom, Egypt; 2 Department of Public Health and Community Medicine, Faculty of Medicine, Tanta University, P.O. Box 31527, El-Gaish Street, Medical Campus, Tanta, Egypt; 3 Batterjee Medical College for Science and Technology, P.O.Box 23819, Prince Abdullah Al-Faisal Street, North Abhor, Jeddah, 21442 Saudi Arabia

**Keywords:** Black fungus, Diabetes Mellitus, Maternal health, Pregnancy, Rural, Prevention

## Abstract

**Background:**

Black fungus (mycoses) is an opportunistic invasive infection that predominantly occurred among immunosuppressed persons. It has been recently detected in COVID-19 patients. The pregnant diabetic woman is susceptible to such infections and needs recognition for protection. This study aimed to evaluate the effect of the nurse-led intervention on the knowledge and preventive practice of diabetic pregnant women regarding fungal mycosis during the COVID-19 pandemic.

**Method:**

This quasi-experimental study was conducted at maternal health care centers in Shebin El-Kom, Menoufia Governorate, Egypt. The study recruited 73 diabetic pregnant women through a systematic random sampling of pregnant women attending the maternity clinic during the period of the study. A structured interview questionnaire was used to measure their knowledge regarding Mucormycosis and COVID-19 manifestations. The preventive practices were assessed through an observational checklist of hygienic practice, insulin administration, and blood glucose monitoring for the prevention of Mucormycosis infection.

**Results:**

The study revealed a statistically significant increment in the participants’ knowledge, preventive practice, personal hygiene, and diabetes self-care scores (9.56 ± 1.75 ,3.6 ± 1.18, 3.18 ± 1.29 post-intervention) comparable to (6.19 ± 1.66, 1.97 ± 1.35, 0.89 ± 1.38  pre-intervention) respectively. There was a significant improvement in the overall COVID-19 protective score against Mucormycosis (from 2.66 ± 1.74 to 4.53 ± 1.43).

**Conclusion:**

Nursing educational sessions had a positive effect on pregnant women’s awareness and preventive behavior. Hence, it is recommended to integrate nurse-led intervention targeting the preventive practice against COVID-19-associated Mucormycosis infection (CAM) as routine services for diabetic pregnant women during antenatal care.

**Supplementary Information:**

The online version contains supplementary material available at 10.1186/s12912-023-01320-x.

## Introduction

The emergence of the novel coronavirus (COVID-19) started at the end of 2019 and is still a global public health threat. COVID-19 is a highly contagious disease with high mortality rates. The excess COVID-19 death is directly attributed to the virus and indirectly to the disrupted basic health services as well as travel restrictions. It is exceptionally higher in patients with chronic medical conditions like diabetes mellitus (DM). Mucormycosis has been evoked as a serious hazard to health during and post COVID-19 infection. It is also called the “Black Fungus”’, which was detected mainly among diabetic patients infected with COVID-19 [1].

Mucormycosis is an invasive fungal infection caused by species of the Mucorales class. The reservoir of infection includes contaminated soil and decaying organic material such as fruit and vegetables. The most virulent pathogenic genera are *Rhizopus*, *Lichtheimia*, and *Mucor* [2].

There is a variation in the clinical presentation of Mucormycosis as it progressively invades the body systems, skin, paranasal sinuses, orbits, lungs, kidneys, central nervous and gastrointestinal systems The pathogenesis induced by these fungal spores relies on two factors: (a) The portal of entry to the body, mainly by respiratory, foodborne followed by contact infection; (b) The medical condition of the infected host. Worldwide, diabetes mellitus is the most significant disease in Mucormycosis infected cases. As well as, immunocompromised (organ transplanted patients and neoplasm)[3]. Pregnancy is considered a state of partial immunosuppression, and several cases of Mucormycosis during pregnancy have been reported. Most cases presented with rhino-cerebral lesions and seldom involvement of the gastrointestinal tract [4].

Globally, the invasive Mucormycosis is more prevalent in India (55.5% ) and the MENA Region; Egypt (17.8%), Iran (9.9%), and Turkey (6.3%) where there is an increase in the number of people suffering from predisposing diseases, especially uncontrolled DM, and the real prevalence of Mucormycosis is still underestimated. Its prevalence is associated with tropical and subtropical climate zones, particularly during the autumn season [5].

Glucocorticoids are used to treat patients with the coronavirus disease through inhibition of the Janus kinase inhibitors or IL-6 receptor inhibitors which induce life-threatening opportunistic infections including invasive mycoses, unexplained immunosuppression and lymphopenia leading to poor outcomes [6].

Moreover, it is thought the SARS Cov 2 virus causes lesions in the tissue of the respiratory airways and the vascular system, which raises the vulnerability of patients to fungal infection. As well as, the rise in blood Ferritin levels during COVID-19 infection provides an enriched medium for the growth and reproduction of the fungus, and undeniably the disordered metabolic profile worsens the prognosis [7].

Recently, there are two published case reports of patients who developed Rhino-orbital Mycosis; proptosis, ophthalmo-plegia, and restricted eye movement; both were COVID-19-infected patients who were suffering from metabolic ketoacidosis and hyperglycemia. One of them died 1 week after admission to the ICU [8, 9].

Previous literature described the epidemiological pattern of Mucormycosis in Egypt of a relatively high incidence rate. Especially with the increased number of patients with uncontrolled blood sugar due to limited healthcare resources directed for DM management. The shortage of surveillance systems, poor awareness of health providers about fungal infections, and lack of communication between physicians, pathologists, and microbiologists in healthcare settings might interfere with identifying the actual incidence of Mucormycosis [10].

Mucormycosis was declared a health emergency, therefore early detection is crucial for the management of the disease. Eradication of Mucormycosis entails controlling risk factors such as observed immunosuppressive drug intake, disordered metabolic profile, lack of neutrophils in the blood, and uptake of desferrioxamine. As well, the proper surgical removal of infected tissue and prompt antifungal therapy are helpful [11].

**Fig. 1:**
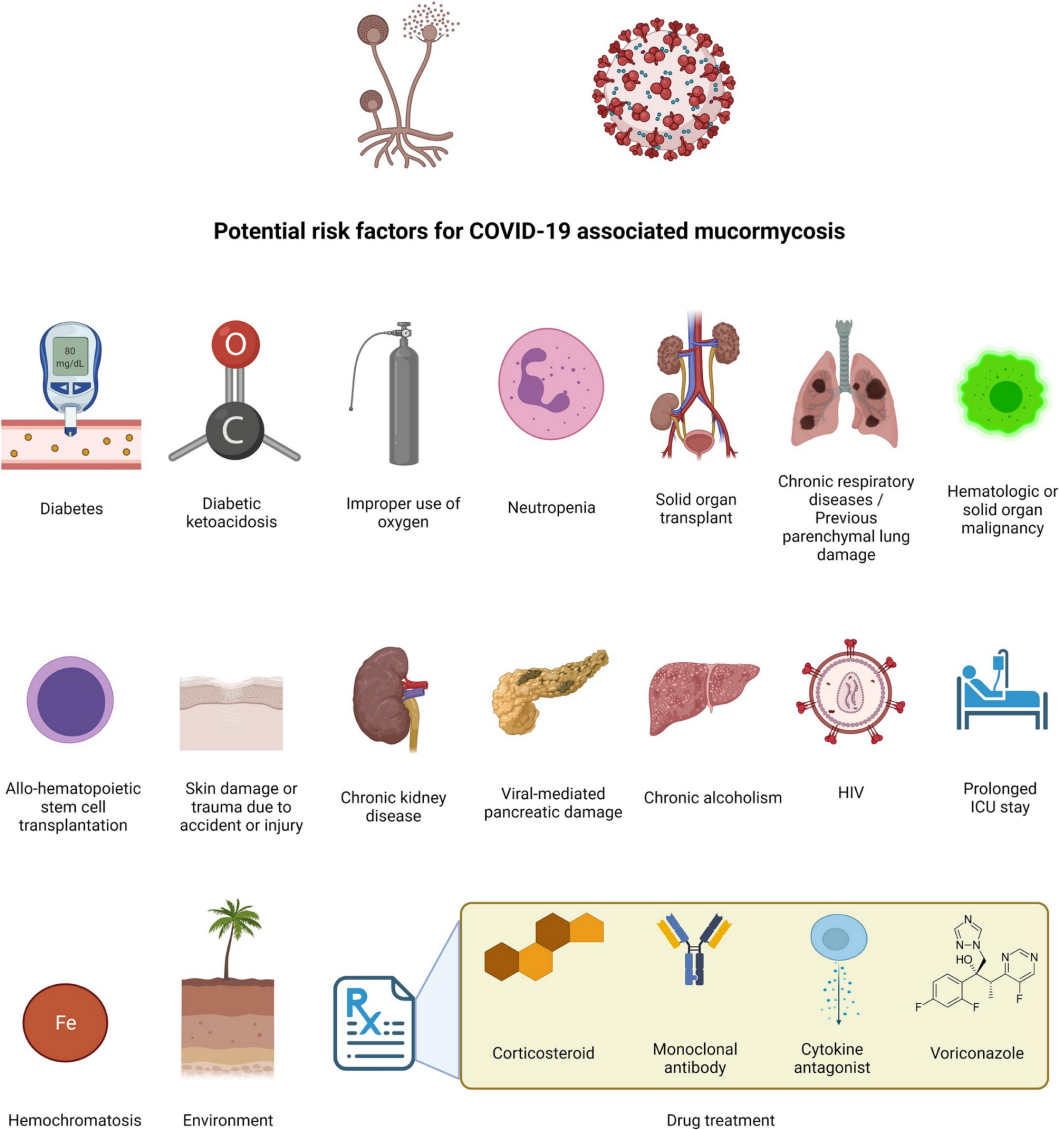
The potential predisposing factors of COVID-19 associated with Mucormycosis quoted from Aranjani et al. 2021 [15] Available at : 10.1371/journal.pntd.0009921.g001

COVID-19 has infected billions of people with 3 million mortalities in more than 200 countries through repeated waves of outbreaks [11]. There has been an increase in Mucormycosis infection rate in COVID-19 patients during repetitive waves [12]. During the first wave, the prevalence of Mucormycosis increased by about 2.1 folds, comparable to the pre- previous year [13]. The documented susceptibility factors for the “Black Fungus” in COVID patients include poor control of blood sugar and prolonged intake of corticosteroids in high doses, as well other potential risk factors as demonstrated in Fig. (1) [13–15].

Nurses are essential healthcare providers since they protect - outpatients against respiratory tract infections and enhance the a sanitary hygienic environment [16]. The prolonged contact nursing staff with patients enables them to improve patients’ knowledge, and attitudes, and acquire competent practices regarding the prevention and control of infectious diseases [17].

The significance of the study is attributed to the relative importance of nursing leadership in health promotion, as reported by the Center for Disease Control and Prevention (CDC) that nursing bodies have a vital role in prevention of aerosol-born infections through directing patients to the precautionary strategies and ensuring their adherence [16]. This study shed the light on the necessity of providing nursing-based programs to recede the susceptibility to Black fungus infection associated with COVID-19 among pregnant diabetic women in rural areas. Especially, there are no specific programs implemented yet in Egypt regarding CAM infection as a part of the maternal health promotion strategies.

The main aim of this study was to evaluate the effect of the nurse-led intervention on knowledge and preventive practice among Egyptian diabetic pregnant women with COVID-19 infection-associated Mucromycosis (CAM) in the rural community. As well as, to improve their perception and behavior to protect themselves against such infection.

### Research hypothesis

The nurse-led intervention regarding (CAM) infection would improve the knowledge level of diabetic pregnant women. Besides, their diabetic self-care and preventive practice against CAM infection would be promoted compared to their prior-intervention level.

## Methods

### Study design

A quasi-experimental design was deployed for this study; a pre and post-interventional study where participants enrolled in five educational sessions. The outcome variables were assessed before and after the intervention.

### Study setting

The study was conducted in Shebin El-Kom, Menoufia Governorate which is located in the Mid Delta Region of Egypt. There are two main Maternal and Child Health (MCH) care centers related to the ministry of health and population. The MCH centers provide integrated maternal and child health services for the population residing within 20 Km surround. Each center receives about 25–35 pregnant females per day, seeking antenatal healthcare services, out of whom about 3 to 4 are diabetic women.

### Sample

A systematic random sampling technique was followed to recruit every 3rd diabetic pregnant woman who fulfilled the inclusion criteria and attended the clinic during the period of the study from March to May 2021.

Number 3 was randomly selected by the researchers using a lottery method. Two days of data collection were determined according to the investigators’ working schedule.

#### The inclusion and exclusion criteria for enrollment of the study participants are shown in Table 1

**Table 1:** Inclusion and exclusion criteria of studied participants

Inclusion criteria	Exclusion criteria
• Diabetic pregnant women aged from 18 to 35 years old with pre-existing diabetes (type 1 or type 2) when admitted to maternity units. • Pregnant women during the first and second trimesters of pregnancy.	• Pregnant women who are diagnosed with mental illness according to their antenatal evaluation and records. • Pregnant women who refused to participate in the study. • Pregnant women who registered for antennal care during the 3rd trimester

#### Sample size calculation

Fischer’s formula below was used to calculate the sample size of the study [18].

$$n = \frac{{{Z^2}P(1 - P)}}{{{d^2}}}$$

**n =** Sample size (where population > 10,000).

**Z =** Normal deviation at the desired confidence interval. In this case, it will be taken at 95%, Z value at 95% is (1.96).

**P =** Proportion of the population with the desired characteristic. Based on the review of literature that examined the same outcomes, the proportion of the population was (0.75) [19].

**Q (1-P) =** Proportion of the population without the desired characteristic.

**d**² = Degree of precision; will be taken to be 10%.

$$\begin{array}{l}n = \frac{{{{(1.96)}^2} \times 0.75(1 - 0.75)}}{{{{(0.1)}^2}}}\\n = 72.03\,{\rm{approximately}}\,{\rm{73}}\,{\rm{participants}}\end{array}$$

#### Pilot study

We conducted a pilot study on 10% of the study sample (8 diabetic pregnant women) of the total sample to ascertain the comprehensibility and applicability of both measuring tools and educational sessions. As well, as the appropriateness of time allocated needed to complete the nurse-led intervention. Regarding the analyzed results of the pilot study, some modifications were performed accordingly.

### Data collection measures and scoring system

The researchers utilized three measuring tools for data collection, which included the followings:

**Measure I**: **An Interviewing Questionnaire**: it was structured by the authors after reviewing similar literature and it is sectioned into 3 parts:

*Part 1*: Included bio-sociodemographic data: woman’s age, telephone number, educational level, and Body Mass Index [20].

*Part II*: Included past and present history of any chronic diseases like hypertension, renal diseases, obesity & thyroid diseases.

*Part III*: Included obstetric history: gravidity, parity, abortion, gestational age in weeks, and regularity of antenatal care.

**Measure II**: **Knowledge Assessment Questionnaire** (Pre & Post-tests):

*Part 1*: Knowledge regarding Mucormycosis based on the Global Guidelines for the Diagnosis and Management of Mucormycosis [21]. It is composed of 6 questions assessing the pregnant women’s knowledge regarding Mucormycosis’ definition, causes, symptoms, risk, factors, complications, and management.

Part II: Knowledge regarding COVID-19 manifestation based on the Centre for Disease Control and Prevention (CDC) Guidelines for Pregnant Women [22]. It assessed the knowledge about maternal signs and symptoms of COVID-19 infection as cough, shortness of breath, an increase in temperature, muscle pain, sore throat, and new loss of taste or smell [23]. Scores were given as follows ; “1” for the correct answer, and “0” for either incorrect and I don’t know answers.

**Measure III**: **Preventive Practice Questionnaire** (Pre & Post-tests):

Part I: Preventive Practice regarding Mucormycosis based on the Global Guidelines for the Diagnosis and Management of Mucormycosis [21]. It included 7 items assessing the diabetic pregnant women’s practice of preventive behavior against Mucormycosis: using masks if you are visiting dusty sites, wearing shoes, putting on long trousers, long sleeve shirts while handling soil (gardening), moss, or manure, wearing gloves when handling materials such as soil, moss, or manure, maintaining personal hygiene including a thorough scrub bath, cleaning skin injuries well with soap and water, especially if they have been exposed to soil or dust and considering warning signs and symptoms of Mucormycosis, Not considering all the cases with a blocked nose as cases of bacterial sinusitis, particularly in the context of immunosuppression and/or COVID-19 patients [24].

*Part I1*: Preventive Practice for Maintenance of Good Diabetes Mellitus Control for Prevention of Mucormycosis based on the Global Guideline for the Diagnosis and Management of Mucormycosis [21]. It included 5 items: regular medical follow-up for diabetes, regular blood glucose measurement, taking prescribed medication, following a prescribed (optimal) diet, and practicing regular physical activity such as walking.

Part III: Preventive practices of COVID-19- precautions for prevention of fungal mycosis based on the Centre for Disease Control and Prevention (CDC) Guidelines for Pregnant Women [22]. It included 9 items: wearing a mask in crowded outdoor settings and with close contact with others who are not fully vaccinated, keeping a safe space between yourself and others (staying at least 6 feet away, which is about 2 arm lengths), avoidance of crowds and poorly ventilated indoor spaces, washing hands or using a hand sanitizer with at least 60% alcohol, avoidance touching eyes, nose, and mouth with unwashed hands, covering coughs and sneezes with a tissue or the inside of your elbow, then wash hands, taking COVID- 19-vaccine, cleaning high touch surfaces regularly or as needed, and monitoring the health status daily e.g. watch for fever, cough, shortness of breath, or other symptoms of COVID-19.

Preventive behavior tool scoring was measured as follows; the items observed to be adequately done were scored “1”, and the items not adequately done or not done were scored “0”.

**Measure IV**: **Direct Observational Checklist for Mucormycosis Preventive Practice**.

Part I: Observational checklist of personal hygienic practices for prevention of COVID-19–associated Mucormycosis (CAM), based on the Centre for Disease Control and Prevention (CDC) Guidelines for pregnant women [22]. It checked for 3 procedures: (a) Handwashing technique (b) Respiratory protection as wearing of cloth face-covering (c) Removal and disposal of respiratory protectors.

Part II: An observational checklist of diabetes control including items related to insulin administration and blood glucose monitoring for diabetic control for Mucormycosis prevention [25].

The items observed to be competent scored “1”, while non-competent or not done were scored “0”. The total score for knowledge and practice of preventive behaviors were summed up and averaged.

### Validity and reliability of the study measures

Face and content validity were performed for the study tools through 5 professional experts in the field of obstetrical nursing. The required modifications were done to ensure the relevance and integrity of the study measures. Test-retest reliability and internal consistency of the study tools were computed using Cronbach’s alpha coefficients. The study measures revealed reliability at Cronbach’s alpha 0.831 for Measure (II), 0.784 for Measure (III), and 0.761 for measure (IV). The tools were translated into Arabic language and reviewed by an Arabic language expert then retranslated into the English language to ensure the accuracy and integrity of the questions.

### Data collection procedures

To be more acquainted with the problem the proper study methodology, the researchers reviewed the current local and international published literature. The practical fieldwork was carried out from the beginning of March to the end of May 2021, related to the outbreak of the third wave of coronavirus and the emergence of fungal Mucormycosis. Each researcher went to the study settings for two days/ week; during the day shift (8.00 am − 2.00 pm). The researchers introduced themselves to the medical and nursing staff members before obtaining administrative approval. The researchers explained the nature and the purpose of the study to the managers and the staff who expressed their full cooperation.

#### The authors conducted the research in the following phases consecutively

5.1. Assessment Phase:
  The researchers met each pregnant woman individually, introduced themselves to the women, and obtained their consent to be enrolled in the study after explaining the purpose of the study. Telephone numbers were taken to facilitate communication and follow-up. We collected their bio-sociodemographic data, obstetric history, and assessed their knowledge level, preventive behavior practices regarding COVID-associated Mucormycosis (CAM), hygienic practice, and diabetes control before the intervention by measures I, II, III, and IV. Each woman’s assessment phase took about 20–25 minutes.
5.2. Planning Phase:
  The researchers prepared educational material based on evidence from literatures [21–30] (Appendix A) covering the following:
  Fungal Mucormycosis : definition, causes, symptoms, risk factors, complications, prevention and management.
  COVID-19 precautions, maternal signs and symptoms of COVID-19 infection.
  Principles of diabetic self-care practice.
    Guiding booklets, pamphlets, and educational videos were prepared in a simple and attractive way to facilitate explanation and to be used as a handout for reference.
5.3. Implementation Phase:
  The researchers conducted the educational sessions according to the participants’ needs derived from the pretest interview. The researchers provide four educational sessions as follows: Knowledge regarding fungal mycosis as well as COVID-19 signs and symptoms (one session). Preventive practices regarding Mucormycotic (three sessions). Each session lasted for 30–40 min. One session was given every week through a face-to-face meeting with 2–3 women, using data show presentation in a well-prepared small hall at the MCH centers. For the woman who missed any session, the researchers repeated the missed one in the week after; when she comes or whenever she comes to complete the intervention. Sessions’ contents and guidelines are provided in Appendix A.
5.4. Evaluation Phase:
  Post-intervention assessment of knowledge level, preventive practices regarding COVID associated Mucormycosis (CAM), hygienic and diabetes control practices was done 3 months after the intervention using measures II and IV.

### Statistical analysis

Data analysis was performed using the IBM Statistical Package for the Social Sciences (SPSS) version 23. Qualitative variables were presented as numbers and percentages, while quantitative ones were described as mean and standard deviation. Paired t-test and ANOVA tests were used to compare the means of normally distributed data. Wilcoxon rank test and Kruskal Wallis test were used instead for non-parametric data. The Mcnemar test was used to evaluate paired categorical variables. Spearman correlation coefficient (_rho_) was used for non- parametric data. *p-value* < 0.05 was considered significant.

## Results

Table 2 showed that the mean age of the study participants was 27.36 ± 1.25 years old, and more than one third attained secondary education level (39%). The mean BMI before pregnancy was 26.74 ± 5.32 kg/m^2^. While the mean gestational age in weeks was 15.77 ± 3.3 and the majority were multipara.

**Table 2:** Bio-Sociodemographic characteristics of the study participants (n = 73 participants)

Variables			N = 73	
			No	%
**Sociodemographic data**	Age in years	- 18–20 - 21–24 - 25–30 - 31–35	4 22 28 19	5.5 30.1 38.4 26.0
		(Mean ± SD)	26.57 ± 4.418	
	Education	- Read & Write - Secondary - University	22 29 22	30.1 39.7 30.1
	BMI (kg/m^2^) before pregnancy	(Mean ± SD)	26.74 ± 5.32	
	Chronic disease other than diabetes	- Yes - No	22 51	30.1 69.9
	If yes, mention this disease?	- Anemia - Hypertension - Renal diseases - Thyroid diseases - Obesity	4 15 0 0 3	5.5 20.5 0.0 0.0 4.1
**Obstetric history**	Gravidity	- 1 - 2 - 3 - > 3	4 28 27 14	5.5 38.4 37.0 19.2
	Parity	- Null para - Multi para	7 66	9.6 90.4
	Abortion	- None - Once or more	60 13	82.2 17.8
	Gestational age in weeks	- 8–12 weeks - 13– 20 weeks	14 59	19.2 80.8
		(Mean ± SD)	15.77 ± 3.30	
	Regularity of antenatal follow-up	- Yes - No	28 45	38.4 61.6

**Fig. 2:**
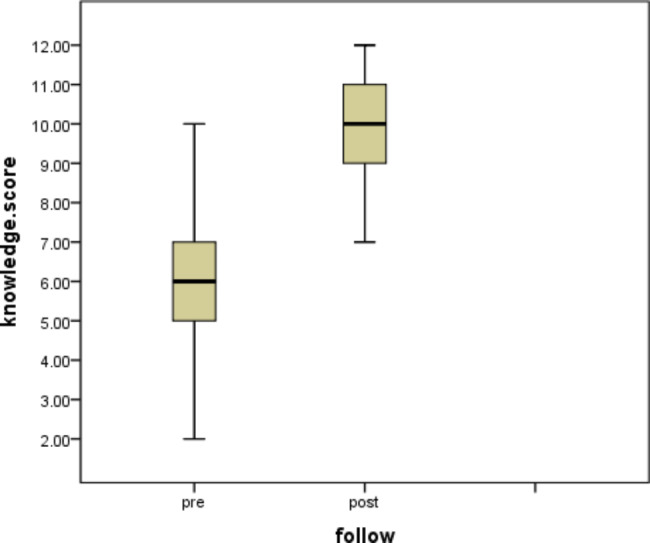
Difference between Pre and Post-intervention of total knowledge score regarding Mucormycosis and COVID 19-Manifestation (n = 73 participants)

Table 3 revealed a highly statistically significant increase in the proportion of correct answers reported by the participants to all knowledge items after the interventions when compared to the pre-intervention responses. The mean knowledge score was increased from 6.19 ± 1.6 pre-intervention to 9.56 ± 1.75 post-intervention (p < 0.001) as shown in Fig. (2).

**Table 3:** Assessment of Knowledge regarding Mucormycosis and COVID-19 manifestation (n = 73 participants)

Variables			Pre Intervention		Three Months Post Intervention		*p-value* ^a^
			No	%	No	%	
**Knowledge regarding Mucormycosis**	The definition of Mucormycosis	- Correct - Incorrect/I don’t Know	15 58	20.5 79.5	51 22	69.9 30.1	< 0.001
	The causes of Mucormycosis	- Correct - Incorrect	12 61	16.4 83.6	43 30	58.9 41.1	< 0.001
	The symptoms of Mucormycosis	- Correct - Incorrect	14 59	19.2 80.8	47 26	64.4 35.6	< 0.001
	The risk factors of Mucormycosis	- Correct - Incorrect	10 63	13.7 86.3	58 15	79.5 20.5	< 0.001
	The complications of Mucormycosis	- Correct - Incorrect	7 66	9.6 90.4	39 34	53.4 46.6	< 0.001
	The management of Mucormycosis	- Correct - Incorrect	13 60	17.8 82.2	51 22	69.9 30.1	< 0.001
**Knowledge about maternal signs and symptoms of COVID-19 infection**	Cough	- Correct - Incorrect	66 7	90.4 9.6	73 0	100 0	0.016
	Shortness of breath	- Correct - Incorrect	53 20	72.6 27.4	63 10	86.3 13.7	0.002
	An increase in temperature	- Correct	73	100	73	100	-
	Muscle pain	- Correct - Incorrect	61 12	83.6 16.4	63 10	86.3 13.7	0.774
	Sore throat	- Correct - Incorrect	63 10	86.3 13.7	66 7	90.4 9.6	0.508
	New loss of taste or smell.	- Correct - Incorrect	65 8	89.0 11.0	71 2	97.3 2.7	0.070
**Total knowledge scores**		Mean ± SD Range	6.19 ± 1.66 2–10		9.56 ± 1.75 4–12		***p-value*** ^***b***^ < 0.001

The total knowledge score is calculated by summation of knowledge answers; where incorrect answer/I don’t know = 0, and correct answer = 1, ^a^ McNemar test, ^b^ Paired t-test.

**Fig. 3:**
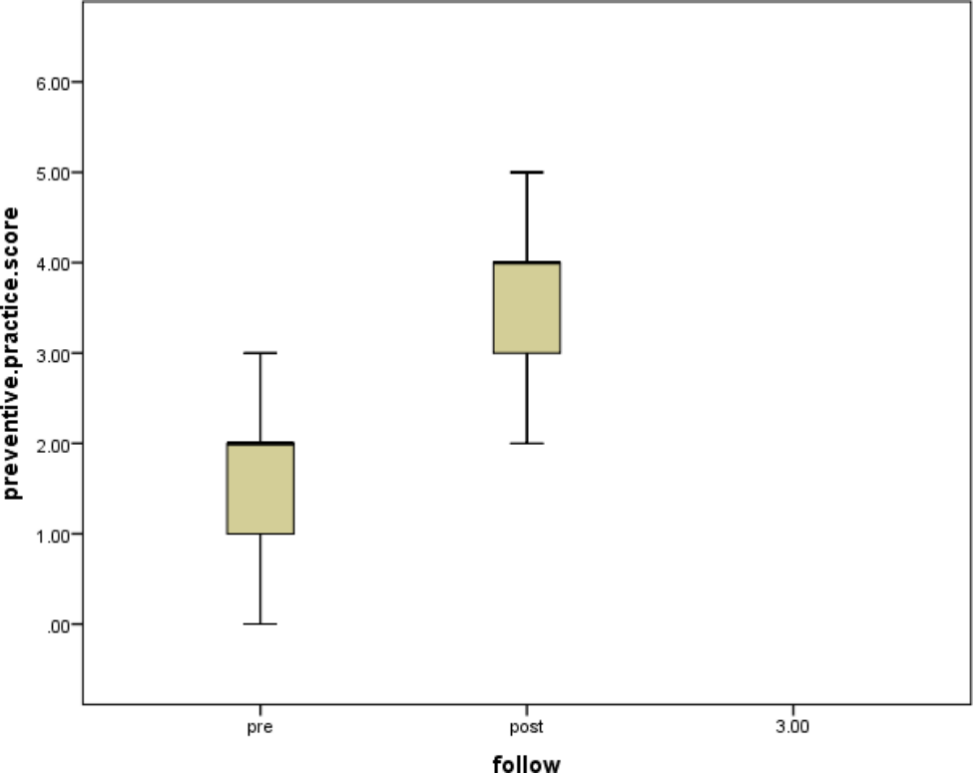
Difference between Pre and Post-intervention of preventive practice behavior score regarding Mucormycosis of the study participants (n = 73 participants)

Table 4 displayed the preventive practice behavior regarding Mucormycosis. The rates of most protective practices after the intervention had significantly increased compared to the reported rates before the intervention (p < 0.001).The mean score for preventive behavior was 1.97 ± 1.35 and statistically improved to 3.60 ± 1.18 post-intervention as shown in Fig. (3).

**Table 4:** Preventive Practice Behavior regarding Mucormycosis (n = 73 participants)

Variables			Pre Intervention		Three Months Post Intervention		*p-value* ^a^
			No	%	No	%	
**Preventive practice behavior regarding Mucormycosis**	Use masks if you are visiting dusty sites.	- Done - Not done	7 66	9.6 90.4	36 37	49.3 50.7	< 0.001
	Wear shoes, long trousers, long sleeve shirts while handling soil (gardening), moss, or manure.	- Done - Not done	43 30	58.9 41.1	47 26	64.4 35.6	0.571
	Wear gloves when handling materials such as soil, moss, or manure	- Done - Not done	11 62	15.1 84.9	49 24	67.1 32.9	< 0.001
	Maintain personal hygiene including a thorough scrub bath	- Done - Not done	7 66	9.6 90.4	11 62	15.1 84.9	0.125
	Clean skin injuries well with soap and water, especially if they have been exposed to soil or dust.	- Done - Not done	64 9	87.7 12.3	72 1	98.6 1.4	0.008
	Taking into consideration warning signs and symptoms of Mucormycosis	- Done - Not done	7 66	9.6 90.4	34 39	46.6 53.4	< 0.001
	Do not consider all the cases with a blocked nose as cases of bacterial sinusitis, particularly in the context of immunosuppression and/or COVID-19 patients	- Done - Not done	5 68	6.8 93.2	14 59	19.2 80.8	0.022
**Preventive practice behavior score**		Mean ± SD Range	1.97 ± 1.35 0–6		3.60 ± 1.18 1–6		***p-value*** ^***b***^ < 0.001

Preventive practice behavior score is calculated by summation of preventive practice behavior answers; where not done/inadequately done = 0, and adequately done = 1,^a^ McNemar test, ^b^ Wilcoxon rank test

As shown in Table 5, there were highly statistically significant improvements in all preventive practices against Mucormycosis (p < 0.001) after the interventions. The overall mean score for preventive practice assessment was improved from 0.89 ± 1.38 pre to 3.18 ± 1.29 post-intervention.

**Table 5:** Diabetes self-care Practice for Prevention of Mucormycosis (n = 73 participants)

Variables			Pre Intervention		Three Months Post Intervention		*p-value* ^a^
			No	%	No	%	
**Personal hygiene assessment for** **prevention** **of Mucormycosis**	Hand washing technique	- Competent - Incompetent	17 56	23.3 76.7	69 4	94.5 5.5	< 0.001
	Respiratory protection as Wearing cloth face covering	- Competent - Incompetent	16 57	21.9 78.1	38 35	52.1 47.9	< 0.001
	Respiratory protection as removal of cloth face covering	- Competent - Incompetent	11 62	15.1 84.9	37 36	50.7 49.3	< 0.001
**Glucose control**	Insulin Administration	- Competent - Incompetent	9 64	12.3 87.7	41 32	56.2 43.8	< 0.001
	Blood glucose Monitoring	- Competent - Incompetent	12 61	16.4 83.6	47 26	64.4 35.6	< 0.001
**Diabetes self-care Mucormycosis Preventive Practice Score**		Mean ± SD Range	0.89 ± 1.38 0–5		3.18 ± 1.29 0–5		***p-value*** ^***b***^ < 0.001

The overall score is calculated by summation of Personal hygiene & Insulin administration score answers; where incompetent = 0, and competent = 1, ^a^ McNemar test, ^b^Wilcoxon rank test

Table 6 displayed a highly statistically significant improvement in most of the items related to good diabetic control for prevention of Mucormycosis after the interventions when compared to the pre-intervention behaviors. The mean overall score of maintenance of good diabetic control was also significantly increased from 2.49 ± 1.25 to 4.00 ± 1.13.

**Table 6:** Practices for maintenance of good diabetic control for prevention of Mucormycosis (n = 73 participants)

Variables			Pre Intervention			Three Months Post Intervention			*p-value* ^*a*^
			No		%	No		%	
**Practices for maintenance of good diabetic control for prevention of Mucormycosis**	Regular medical Follow up for diabetes	- Done - Not done	66 7	90.4 9.6		70 3	95.9 4.1		0.344
	Regular blood glucose measurement	- Done - Not done	11 62	15.1 84.9		58 15	79.5 20.5		< 0.001
	Taking Prescribed medication	- Done - Not done	66 7	90.4 9.6		70 3	95.9 4.1		0.289
	Tacking prescribed(optimal) diet	- Done - Not done	28 45	38.4 61.6		49 24	67.1 32.9		< 0.001
	Practicing regular physical activity as waking	- Done - Not done	11 62	15.1 84.9		45 28	61.6 38.4		< 0.001
**Score of Practices for Maintenance of Good Diabetic Control for Prevention of Mucormycosis**		Mean ± SD Range	2.49 ± 1.25 0–5			4.00 ± 1.13 1–5			***p-value*** ^***b***^ < 0.001

The overall score is calculated by summation of practices for maintenance of good diabetic control answers; where not done answer = 0, and done answer = 1. ^a^ McNemar test, ^b^Wilcoxon rank test

Table 7 indicated a highly statistically significant improvement in most of the precautionary items especially avoidance of crowds and regular hand washing (from 26%, 28.8% to 69.9%, 67.7% respectively). The overall mean score of practices related to COVID-19 precautions significantly increased from 2.66 ± 1.74 pre to 4.53 ± 1.43 post-intervention.

**Table 7:** COVID-19 Precautions for Prevention of Mucormycosis associated infection (n = 73 participants)

Variables			Pre Intervention		Three Months Post Intervention		*p-value* ^a^
			No	%	No	%	
**COVID 19 Precautions for Mucormycosis Prevention**	Wear a mask in public when you interact with other people	- Done - Not done	23 50	31.5 68.5	36 37	49.3 50.7	0.015
	Keep a safe space between yourself and others (stay at least 6 feet away, which is about 2 arm lengths).	- Done - Not done	12 61	16.4 83.6	15 58	20.5 79.5	0.581
	Avoid crowds and poorly ventilated indoor spaces.	- Done - Not done	19 54	26.0 74.0	51 22	69.9 30.1	< 0.001
	Wash your hands. If soap and water are not available, use a hand sanitizer with at least 60% alcohol.	- Done - Not done	21 52	28.8 71.2	56 17	76.7 23.3	< 0.001
	Avoid touching your eyes, nose, and mouth with unwashed hands.	- Done - Not done	22 51	30.1 69.9	26 47	35.6 64.4	0.541
	Cover coughs and sneezes with a tissue or the inside of your elbow. Then wash your hands.	- Done - Not done	46 27	63.0 37.0	58 15	79.5 20.5	0.045
	Taking COVID- 19-vaccine	- Done - Not done	0 73		0 73		**-**
	Clean frequently touched surfaces daily using household cleaners, such as soap or detergent	- Done - Not done	24 49	32.9 67.1	49 24	67.1 32.9	< 0.001
	Monitor your health daily e.g. Watch for fever, cough, shortness of breath, or other symptoms of COVID-19.	- Done - Not done	27 46	37.0 63.0	40 33	54.8 45.2	0.021
**The score of COVID-19 Precautions for Mucormycosis Prevention**		Mean ± SD	2.66 ± 1.74		4.53 ± 1.43		***p-value*** ^**b**^ < 0.001

The score is calculated by summation of COVID 19 precautions answers; where not done answer = 0, and done answer = 1 ^a^ McNemar test, ^b^ Wilcoxon rank test

Table 8 indicated a significant negative correlation between sociodemographic data of participants and total knowledge score regarding COVID-19 associated Mucromycosisis (CAM) before the intervention. While, there was a negative insignificant correlation between sociodemographic data of participants and preventive practice behavior regarding (CAM) pre and post the intervention.

**Table 8:** Relationship between total Knowledge Score, Preventive Practice Behavior Score, and Sociodemographic Data of the study participants Pre & Post the Intervention (n = 73 participants)

Variables		Total Knowledge score				Practice behavior score			
		Pre-Intervention		Post -Intervention		Pre-Intervention		Post-Intervention	
		Mean ± SD	Test of sig. *p-value*	Mean ± SD	Test of sig. *p-value*	Mean ± SD	Test of sig. P-value	Mean ± SD	Test of sig. (P-value)
**Age** **(years)**	18–20 21–24 25–30 31–35	6 ± 0 6.09 ± 1.69 6.54 ± 1.89 5.84 ± 1.42	F^a^= 0.715 0.547	9.25 ± 0.96 9.68 ± 2.42 9.54 ± 1.55 9.53 ± 1.26	F^a^=0.079 0.971	2.04 ± 1.14 1.59 ± 0.59 4.5 ± 1.0 1.79 ± 1.78	KW^b^=12.2 0.007	4.25 ± 0.5 3.55 ± 0.96 3.64 ± 1.09 3.47 ± 1.58	KW ^b^= 1.7 0.635
**Education**	Read &write Secondary University	6.0 ± 1.54 6.21 ± 1.59 6.36 ± 1.92	F^a^= 3.736 0.029	10.18 ± 1.18 9.34 ± 2.11 9.23 ± 1.60	F^a^=1.934 0.152	1.45 ± 0.74 1.86 ± 1.39 2.45 ± 1.55	KW^b^=5.37 0.02	3.41 ± 0.96 3.36 ± 1.47 3.93 ± 1.03	KW^b^= 2.96 0.085
***Spearman Correlation coefficient r (p-value)***		r= -0.239 (0.042)		r= -0.228 (0.052)		r= -0.059 (0.619)		r = -0.085 (0.474)	

^a^ F : ANOVA, ^b^ KW: Kruskal-Wallis test

## Discussion

Coinciding the novel coronavirus disease (COVID-19) pandemic, the cases of COVID-19-Black fungus co-infections have been increasingly reported worldwide. Recent literature reported that the vast majority of CAM patients suffered from at least one medical comorbidity; diabetes mellitus is the main contributing diseases (79.1%) [31].

The present study enrolled 73 diabetic pregnant women, who were assessed for their knowledge level and if they follow the preventive behavioral practice against COVID-19 asscioated Mucromycosisis infection (CAM). The recruited participants received 3 weeks of nurse-led educational sessions, then they were reassessed to determine the effectiveness of this intervention.

In the pre-intervention phase, this study revealed an acceptable knowledge level among pregnant women, because of the persistent dissemination of COVID-19 related information by the Egyptian Ministry of Health and Population (MOHP), community organizations and people through their social accounts [32]. The observed acceptable knowledge about COVID-19 manifestation, complications, and preventive measures was replicated through enormous studies with different targeted study subjects [33, 34].

Meanwhile, the detected low score of proper preventive practices might be attributed to several factors including insufficient face masks, unavailability of soap for handwashing in public places, the increased expense of hand gel sanitizers, barriers against physical/ social distancing, challenges against vaccination, and shortage of material preparedness in face of the crisis [35].

### Knowledge Assessment regarding COVID 19-with mucormycosis infection (CAM)

The current study findings revealed a significant enhancement in knowledge level when compared to the pre-intervention scores. This finding was in the same line with Zhang et al., 2020 who concluded that health education regarding the prevention of infectious disease is potentially essential [36]. Likewise, a recent study involved pregnant women in Ghana reported that women who received awareness sessions about COVID-19 in health facilities prompted an ample level of knowledge (76.2%) about COVID-19 disease and its safety measures compared to those who didn’t [37].

These results were agreed with Kumbeni, 2021 who found that the prevalence of adequate knowledge about COVID-19 was 85.6%, and added that receiving COVID-19 education at healthcare settings was positively associated with adequate knowledge of COVID-19 [38]. Meanwhile, the current study findings are in contrast with Queensland Clinical Guidelines, (2020) which reported that a large proportion of pregnant women were unaware of COVID-19 clinical presentation and precautionary guidelines [39].

Noted that there was a remarkable high correctness of the information regarding Mucormycosis in the post-intervention analysis, this probably due to already lacked awareness and the shortage of information regarding Mucromycosisis. While the relative increase in knowledge related to the COVID-19 manifestation is attributed to the existed good awareness level as discussed before.

### Preventive practice behavior regarding mucormycosis

Our pre-posttest analysis found a significant increased adherence of participants to preventive practices against Mucormycosis including “Wearing masks when visiting dusty areas” ( 9.6–49.3%), “Wearing gloves when handling materials such as soil, moss, or manure” (15.1–67.1%), “Cleaning skin injuries well, especially if they have been exposed to soil or dust”.(87.7–98.6%) and “Considering warning signs and symptoms of Mucormycosis” (9.6–46.6%). Our findings are consistent with a similar study in India, which displayed that the precautionary measures were better followed by the immunosuppressed patients to avoid any black fungus infection scenario after receiving awareness sessions [40].

### Practices for maintenance of good diabetic control for prevention of mucormycosis

Similar literature mentioned that educational intervention led by nursing staff for women with gestational diabetes leads to enhancement of their health practices and favourable pregnancy outcomes [41]. The present study unveiled a significant increase in the number of women who competently administered the required insulin doses and monitored their blood sugar regularly after the intervention [pre (12.3%,16.4%) to post (56.2%,64.4%)] respectively.

In support of our results, a previous study reported a significant reduction in the postprandial blood sugar (PPBS) level and insulin dose among pregnant women with gestational diabetes mellitus (GDM) after receiving maternal nursing health education sessions [42]. Furthermore, a previous study reported a significant increase in the proportion of women who developed good diabetic self-care practice after receiving awareness sessions for (GDM) management (pre 6.7%, post 18.3%) and improvement in all self-care activities subscales [43]. The observed improved control practices are helpful in the prevention of Mucormycosis, which necessitates an optimum level of awareness about metabolic control of diabetes mellitus [44].

### COVID-19 precautions for mucormycosis prevention

The present study showed a remarkable improvement in the pregnant women’s practice, as the mean score of COVID-19 precautionary practice was 4.53 ± 1.43 post-intervention, compared to 2.66 ± 1.74 pre-intervention. This indicates the effectiveness of the performed nursing intervention sessions in promoting the women’s preventive practice. Consistently, Fikadu et al., 2021 who studied the practices and knowledge of pregnant women regarding COVID-19 prevention in Guraghe Zone Hospitals demonstrated that COVID-19 preventive measure practice of women who were regularly visiting the hospital was 54.84% and attributed their findings to the efficacious intervening measures provided by the government and health institution [37]. These observations are also supported by Hassan et al.2020, who concluded that improving the pregnant women’s knowledge regarding COVID-19 through health education, resulted in the enforcement of their self-protective measures against COVID-19 infection [45].

### Mediators associated with knowledge of COVID-19 and COVID-19 preventive practices

Upon investigating the underpinnings related to knowledge and practice level, we detected no association between age and knowledge score either in the pre or post-intervention assessment. While high knowledge score was detected among participants who attained higher university education, in the pre-intervention assessment only. These observations were replicated by Kumbeni et al., 2021 in Ghana, who found that females with secondary or higher education had 3.40 and 10.61 odds respectively, of having satisfactory knowledge about COVID-19 comparable to their peers with no formal education [38].

Empirical research investigated the integrational effect of compulsory education on the mothers’ health attitudes and behaviors, it showed that women who attained at least a primary education might be more exposed to health information and subsequently more likely to assume preventive measures to avoid getting infected with the disease [46].

The analysis also, brought up that women aged between 25 and 30 years were significantly more engaged in acceptable COVID-19 preventive practices before the intervention, as well as those with an advanced level of education (mean score 4.5 ± 1 p = 0.007 and 2.45 ± 1.55 p = 0.02 respectively). Consistently, a related study found that women aged 28 years old and above; with secondary /university education had an odds of 2.12 and 4.11 times respectively of practicing preventive behavior against COVID-19 infection than their counterparts [38].

Previous literature has reported that older age is a liability agent for serious complications and mortality related to COVID-19 infection [47, 48]. This might explain why older pregnant women in the present study had a higher score for COVID-19 preventive practices to avoid this infection due to consternation from its consequences.

Likely, the significant association between good preventive behavioral scores between women in older age groups and higher educational attainment is explained by the authors that those women are privileged to have healthier attitudes and behaviors than their counterparts.

Correlational analysis unveiled a weak negative significant correlation (r=-0.22, p = 0.04) between the knowledge level and demographic characteristics in the pre-intervention phase. That might be owing to the existed shortage of information regarding COVID-19 associated with Mucormycosis(CAM).

However, this observed discrepancy ceased in the post-evaluation phase, since this correlation was concealed after receiving the intervention which indicates the successfulness and effectiveness of our nurse-led intervention to deliver the health message to the targeted audience of varied demographic characteristics.

## Conclusion

This study showed that there was an improvement in the diabetic pregnant women’s knowledge regarding COVID-19 associated Muycormycosis (CAM) post the intervention than pre the intervention. Furthermore, there was an enhancement in the diabetic pregnant women’s preventive practice against (CAM) post the intervention, which support the research hypothesis. Overall, the study findings confirm the substantial role that nursing staff plays in providing information regarding COVID-19 associated Muycormycosis infection at antenatal care settings.

## Recommendation

It is recommended to conduct further research within different contexts to measure the efficacy of such an intervention. As well as, to implement a nurse-led educational program to the healthcare community about black fungus associated with COVID Pandemic, counseling to diabetic pregnant women, and develop home messages to pregnant women receiving antenatal care about this associated infection.

## Relevance to clinical practice

Since diabetic pregnant women are vulnerable to Black fungus infection and susceptible to severe COVID-19-related complications, they are crucially needed to promote their awareness and preventive practice. The initial implications of this study were effective. Therefore, health care organizations should develop a comprehensive strategy for diabetic pregnant women especially in rural areas to enhance their awareness and resistance against infectious diseases, particularly COVID-19 associated Muycormycosis (CAM), through continuing education and mentoring programs.

## Strengths and limitations

Our study is the first research that deployed the nurse-led intervention to improve the knowledge and preventive behavior of pregnant diabetic women. Our findings are promising to inform the responsible health authorities to channel resources towards the nursing role during the fight against COVID-19 associated with Mucoromycosis (CAM). The cross-sectional nature of our study wouldn’t help to infer causality. The self-reported method of data collection might expose the data to the possibility of recall bias. Also, owing to the lack of funding and supportive resources, the sample size was relatively small which doesn’t allow for the generalization of findings to the entire community of pregnant diabetic women in our country, Egypt.

## Electronic supplementary material

Below is the link to the electronic supplementary material.

Appendix A: Contents of Educational Sessions

## Data Availability

The datasets generated and analyzed during the current study is available from the corresponding author on a reasonable request.

## References

[1] Zhou F, Yu T, Du R (2020). Clinical course and risk factors for mortality of adult in patients with COVID-19 in Wuhan, China: a retrospective cohort study. Lancet.

[2] Skiada A, Pavleas I, Drogari-Apiranthitou M (2020). Epidemiology and diagnosis of mucormycosis: an update. J. Fungi.

[3] Jeong W, Keighley C, Wolfe R, Lee WL. Slavin, MA, Kong DC et al. The epidemiology and clinical manifestations of mucormycosis: a systematic review and meta-analysis of case reports. Clin. Microbiol. Infect. 2019; (25): 26–34. [Google Scholar] [CrossRef][Green Version]. 10.1016/j.cmi.2018.07.011 30036666

[4] Rithvik R, Vengadakrishnan K (2015). Gastric mucormycosis presenting as perforated gastric ulcer in pregnancy: a Case Report. IJSS Case Reports and Reviews.

[5] Stemler J, Hamed K, Salmanton-García J, Rezaei-Matehkolaei A, Gräfe SK, Sal E (2020). Mucormycosis in the Middle East and North Africa: analysis of the FungiScope® registry and cases from the literature. Mycoses.

[6] Rodriguez-Morales AJ, Sah R, Millan-Oñate J, Gonzalez A, Montenegro-Idrogo JJ, Scherger S, Franco-Paredes C, Henao-Martínez AF. COVID-19 associated mucormycosis: the urgent need to reconsider the indiscriminate use of immunosuppressive drugs. Ther Adv Infect Dis. 2021 Jun 18;8:20499361211027065. DOI: 10.1177/20499361211027065. PMID: 34211710; PMCID: PMC8216396. 10.1177/20499361211027065 PMC8216396 34211710

[7] Gokulshankar SI, Mohanty BK (2021). COVID-19 and black fungus. Asian Journal of Medicine and Health Sciences.

[8] Sargin F, Akbulut M, Karaduman S, Sungurtekin H (2021). Severe rhinocerebral mucormycosis case developed after COVID 19. J Bacteriol Parasitol.

[9] Waizel-Haiat S, Guerrero-Paz JA, Sanchez-Hurtado L (2021). A case of fatal rhino-orbital mucormycosis associated with new onset diabetic ketoacidosis and COVID-19. Cureus.

[10] Zaki SM, Elkholy IM, Elkady NA, Abdel-Ghany K. Mucormycosis in Cairo, Egypt: a review of 10 reported cases. Med Mycol. 2014;52(1):73–80. DOI: 10.3109/13693786.2013.809629. PMID: 23848229. 10.3109/13693786.2013.809629 23848229

[11] Kasapoglu F, Coskun H, Ozmen OA, Akalin H, Ener B.Acute invasive fungal rhinosinusitis: evaluation of 26 patients treated with endonasal or open surgical procedures. Otolaryngol Head Neck Surg.2010; 143:614–620. 10.1016/j.otohns. 2010. 08.017. 10.1016/j.otohns.2010.08.017 20974328

[12] Song G, Liang G, Liu W. Fungal co-infections associated with global COVID-19 pandemic: a clinical and diagnostic perspective from China. Mycopathologia. 185(4):599–606.doi: 10.1007/s11046-020-00462-9. 10.1007/s11046-020-00462-9 PMC7394275 32737747

[13] Gandra S, Ram S, Levitz SM (2021). The “Black Fungus” in India: the emerging Syndemic of COVID-19-Associated Mucormycosis. Ann Intern Med.

[14] Patel A, Agarwal R, Rudramurthy SM, Shevkani M, Xess I, Sharma R, Savio J, et al. Multicenter Epidemiologic Study of Coronavirus Disease-Associated Mucormycosis, India. Emerg Infect Dis. 2021;27(9): 2349–2359. DOI: 10.3201/eid2709.210934. Epub ahead of print. PMID: 34087089. 10.3201/eid2709.210934 PMC8386807 34087089

[15] Aranjani JM, Manuel A, Abdul Razack HI, Mathew ST (2021). COVID-19–associated mucormycosis: evidence-based critical review of an emerging infection burden during the pandemic’s second wave in India. PLOS Neglected Tropical Diseases.

[16] Center for Diseases Control and Prevention (CDC). Prevention Strategies for Seasonal Influenza in Healthcare Settings. Saving lives protecting people, 2016. Available at: http://www.cdc.gov/flu/professionals/infectioncontrol/healthcaresettings.htm. [Date accessed: 16 June 2016].

[17] Porto JS and Marziale MH. Reasons and consequences of low adherence to standard precautions by the nursing team. Rev Gaucha Enferm. 2016 Jun;37(2):e57395. English, Portuguese. DOI: 10.1590/1983-1447.2016.02.57395. Epub 2016 May 31. Erratum in: Rev Gaucha Enferm. 2016 Jun;37(2):e57395x. PMID: 27253597. 10.1590/1983-1447.2016.02.57395 27253597

[18] Fischer’s Formula for sample size calculation available at. https://www.coursehero.com/file/p75sn16/10-372-SAMPLE-SIZE-DETERMINATION-The-Fischers-formula-below-was-used-to/). Acessed in January 2021.

[19] Corzo-León DE, Chora-Hernández LD, Rodríguez-Zulueta AP, Walsh TJ. Diabetes mellitus as the major risk factor for Mucormycosis in Mexico: Epidemiology, diagnosis, and outcomes of reported cases. Med Mycol. 2018 Jan 1;56(1):29–43. doi: 10.1093/mmy/myx017. PMID: 28431008. 10.1093/mmy/myx017 28431008

[20] World Health Organization (WHO). Mean Body Mass Index (BMI)”. World Health Organization. Retrieved 5 February 2019. Available at: https://www.who.int/data/gho/data/themes/topics/topic-details/GHO/ncd-risk-factors.

[21] Cornely OA, Alastruey-Izquierdo A, Arenz D, Chen SCA, Dannaoui E, Hochhegger B, Mucormycosis, ECMM MSG Global Guideline Writing Group, in cooperation with the Mycoses Study Group Education and Research Consortium (2019). Global guideline for the diagnosis and management of mucormycosis: an initiative of the european Confederation of Medical Mycology. Lancet Infect Dis.

[22] Centers for Disease Control and Prevention) CDC(Guidelines for Pregnant Women, (2021). Available at: https://www.cdc.gov/coronavirus/2019-ncov/need-extra-precautions/pregnant-people.html.

[23] Shanin MA, Metwally HM, Desoky MM, Abd Elaziz SM, Mohamed RA, Hassan GA. Effect of structured postpartum nursing intervention involving COVID-19 Precautionson Mother’s knowledge, practice, fear level and neonatal care. SYLWAN,2021; 165(6)21:48.

[24] Daitkar SA, Andhale AS, Waghmare SA, KambleHE Type of Manuscript: Review Mucormycosis Associated With COVID-19: A Review (Coronavirus: Mucormycosis Cases Spike In COVID-19 Recovered Patients). IJSART,2022; 8 (3)7:14. ISSN [ONLINE]: 2395 – 1052 ].

[25] American Diabetes Association’s Standards of Medical Care in Diabetes—2020. Diabetes Care 2020;43(Suppl. 1): S1–S212. The complete 2020 Standards supplement, including all supporting references, is available at professional.diabetes.org/standards. 10.2337/cd20-as01.

[26] Screening, Diagnosis & Management of Mucormycosis. available at : https://vikaspedia.in/health/health-campaigns/COVID-management/screening-diagnosis-management-of-Mucormycosis. Accessed in December 2020.

[27] The Indian Council of Medical Research (ICMR), The ICMR-health ministry advisory (8 April 2020). Department of Science & Technology (DST) approves funding for developing a gel for nasal passage as prevention for COVID 19”. Press Information Bureau, Government of India Press Information bureau. Retrieved 13 April 2020. Available at: https://pib.gov.in/PressReleseDetail.aspx?PRID=1612161.

[28] Davis CP. Facts you should know about Mucormycosis (zygomycosis). infectious disease health center/Mucormycosis center, Medicine Net’s General Health Newsletter.2021 https://www.medicinenet.com/Mucormycosis/article.htm.

[29] National Institute of Diabetes and Digestive and Kidney Diseases. Pregnancy if You Have Diabetes. Pregnancy if you have diabetes. Available at: https://www.niddk.nih.gov/health-information/diabetes/diabetes-pregnancy.

[30] Pregnancy, birth and newborn care. Available at: https://www.nationalwomenshealth.adhb.govt.nz/womens-health-information/maternity/ Accessed in February 2022.

[31] Riad A, Shabaan AA, Issa J, Ibrahim S, Amer H, Mansy Y, Kassem I, Kassem AB, Howaldt H-P, Klugar M, Attia S (2021). COVID-19-Associated Mucormycosis (CAM): case-series and global analysis of mortality risk factors. Journal of Fungi.

[32] Saied AA, Metwally AA, Madkhali NAB, Haque S, Dhama K (2021). Egypt’s COVID-19 recent happenings and perspectives: a Mini-Review. Front Public Health.

[33] Nwafor JI, Aniukwu JK, Anozie BO, Ikeotuonye AC, Okedo-Alex IN (2020). Pregnant women’s knowledge and practice of preventive measures against COVID-19 in a low-resource african setting. Int J Gynecol Obstet.

[34] Al-Hanawi MK, Angawi K, Alshareef N, Qattan AMN, Helmy HZ, Abudawood Y, et al. Knowledge, Attitude and Practice Toward COVID-19 Among the Public in the Kingdom of Saudi Arabia: A Cross-Sectional Study. Front Public Heal. 2020; 8:217. 10.3389/fpubh.2020.00217 PMID:32574300. 10.3389/fpubh.2020.00217 PMC7266869 32574300

[35] World Health Organization (WHO). Interim Recommendations 1 April 2020 [Internet]. 2020 [cited 2020 Oct 24]. https://www.who.int/docs/default-source/inaugural-who-partners-forum/who-interim-recommendationon-obligatory-hand-hygiene-against-transmission-of-COVID-19.pdf.

[36] Zhang X, Sun Y, Ye D, Sun Z, Su H, Ni J (2020). Analysis on mental health status of community residents in Hefei duringSARS spread. Chin J Dis Contr Prev.

[37] FikaduY Yeshane A, Melis T (2021). COVID-19 preventive measure Practices and knowledge of pregnant women in Guraghe Zone Hospitals. International Journal of Women’s Health.

[38] Kumbeni MT, Apanga PA, Yeboah EO, Lettor IK (2021). Knowledge and preventive practices towards COVID-19 among pregnant women seeking antenatal services in Northern Ghana. Plos one.

[39] Queensland Clinical Guidelines Translating evidence into best clinical practice (COVID-19 and pregnancy. Perinatal care of suspected or confirmed COVID19 pregnant mother. Guideline No. MN20.63-V3-R25. X.Available at: https://www.health.qld.gov.au/qcg.

[40] Singh K, Kumar S, Shastri S, Sudershan A, Mansotra V (2022). Black fungus immunosuppressive epidemic with COVID-19 associated mucormycosis (zygomycosis): a clinical and diagnostic perspective from India. Immunogenetics.

[41] Mendelson SG, McNeese-Smith D, Koniak-Griffin D, Nyamathi A, Lu MC. A community-based parish nurse intervention program for Mexican American women with gestational diabetes. J Obstet Gynecol Neonatal Nurs. 2008;37(4):415 – 25. DOI: 10.1111/j.1552-6909.2008.00262.x. PMID: 18754979. 10.1111/j.1552-6909.2008.00262.x 18754979

[42] Viswanath, Lekha & Jose, Anne. (2015). Impact of a Comprehensive Nursing Intervention Package on the Glycemic Control of Women with GDM. Abstract, Journal of Nurse-Midwifery and Maternal Health.2015; 1 (1):1. Available from: https://www.researchgate.net/publication/343375904_Impact_of_a_Comprehensive_Nursing_Intervention_Package_on_the_Glycemic_Control_of_Women_with_GDM [accessed Jan 07 2022].

[43] Saboula NE, Ahmed NA, Rashad RH (2018). Effect of nursing intervention on knowledge, attitude and self-care activities among gestational Diabetic Women. International Journal of Novel Research in Healthcare and Nursing.

[44] Slavin M and Thursky K, What is Mucormycosis, the fungal infection affecting COVID patients in India?BBC NEWS,India, May 13, 2021. Available at: https://theconversation.com/what-is-Mucormycosis-the-fungal-infection-affecting-COVID-patients-in-india-160707.

[45] Hassan AZ, Elsayed DM, Abosree TH, Eltohamy NA (2020). Pregnant women’s knowledge, attitude and self-protective measures practice regarding Corona virus prevention: Health Educational intervention. Egyptian Journal of Health Care.

[46] Feinstein BL, Sabates R, Anderson TM, Sorhaindo A, Hammond C. What are the effects of education on health? In Desjardins R and Schuller T(Eds.) *Measuring the Effects of Education on Health and Civic Engagement* 2006 (pp:171–354).Center for education and research innovation, Proceedings of the Copenhagen Symposium.

[47] Fei Z, Ting Y, Ronghui D, Guohui F, Ying L, Zhibo L, et al. Clinical course and risk factors for mortality of adult inpatients with COVID-19 in Wuhan, China: a retrospective cohort study. Lancet. 2020; 395(10229):1054–1062. 10.1016/S0140-6736(20)30566-3 PMID: 32171076. 10.1016/S0140-6736(20)30566-3 PMC7270627 32171076

[48] Wu C, Chen X, Cai Y, Xia J, Zhou X, Xu S (2020). Risk factors Associated with Acute respiratory distress syndrome and death in patients with Coronavirus Disease 2019 Pneumonia in Wuhan, China. JAMA Intern Med.

