# Supplementary material for: Effect of nurse-led intervention on knowledge and preventive behavior of diabetic pregnant women regarding COVID-19 associated mucromycosis infection in mid-delta region of Egypt
Source: BMC Nurs. 2023 May 22;22:175. doi: 10.1186/s12912-023-01320-x (PMC10200695; doi:10.1186/s12912-023-01320-x)
Supplement: Supplementary file 1 — Appendix A: Contents of Educational Sessions [file 12912_2023_1320_MOESM1_ESM.docx]

**Appendix A**

**Contents of Educational Sessions**

1. **The First Session**

**Part 1:** Each diabetic pregnant woman received an individualized and comprehensive awareness session about Mucormycosis infection based on the Global Guideline for the Diagnosis and Management of Mucormycosis**.** This part explored the following points:

- **Definition:** Mucormycosis is a fungal infection that mainly affects people who are on medication for other health problems that reduce their ability to fight environmental pathogens.
- **Causes:** Mucormycosis is caused by a group of fungi known as Mucormycetes which are present everywhere in the environment.
- **Risk Factors as stated by the ICMR-health ministry advisory, 2020:**
- Uncontrolled diabetes mellitus
- Immunosuppression by steroids
- Prolonged ICU stays.
- Co-morbidities – post-transplant/malignancy
- Voriconazole therapy
- **Symptoms as stated by the ICMR-health ministry advisory, 2020:**
- Pain and redness around the eyes and/or nose
- Fever
- Headache
- Coughing
- Shortness of breath
- Bloody vomits.
- Altered mental status
- **Complications:**
- Blindness
- Meningitis
- Brain abscesses
- Osteomyelitis
- Pulmonary hemorrhages
- Gastrointestinal hemorrhages
- Cavitary lesions in organs and eventually secondary bacterial infections, sepsis, and death
- **Management**:
- Control diabetes and diabetic ketoacidosis.
- Reduce steroids (if the patient is still on) intending to discontinue rapidly.
- Discontinue immunomodulation drugs.
- No antifungal prophylaxis is needed.
- Extensive Surgical Debridement - to remove all necrotic materials.
- Medical treatment: maintain adequate systemic hydration, antifungal therapy, for at least 4-6 weeks, monitor patients clinically and with radio-imaging for response and to detect disease progression.

**Part II:** Each diabetic pregnant woman received an individualized and comprehensive awareness session regarding COVID- 19 include the following items:

- **Maternal signs and symptoms of COVID-19** : -
- Cough
- Shortness of breath
- An increase in temperature
- Muscle pain
- Sore throat
- New loss of taste or smell
- Warning signs for risky pregnancy

1. **The Second Session**

**Part I:** Each diabetic pregnant woman was taught about practices of preventive behaviours regarding Mucormycosis. It included the following practices:

- Use masks if you are visiting dusty sites
- Wear shoes, long trousers, long sleeve shirts, and gloves while handling soil (gardening), moss or manure
- Maintain personal hygiene including thorough scrub bath
- Clean skin injuries well with soap and water, especially if they have been exposed to soil or dust
- Control hyperglycemia
- Control diabetes by monitoring blood glucose and 6- Use antibiotics/anti-fungal judiciously)
- Do not miss warning signs and symptoms,
- Do not consider all the cases with a blocked nose as cases of bacterial sinusitis, particularly in the context of immunosuppression and/or COVID-19 patients
- Do not hesitate to seek aggressive investigations, as appropriate.

**Part II.** Each diabetic pregnant woman was taught practices of good diabetic control for prevention of Mucormycosis. It included the following practices:

- Taking optimal diet**:**  Eating three small to moderate size meals and three snacks per day is appropriate. Overall, the dietary composition is usually recommended as 18 - 20% protein, < 10% saturated fat, < 10% polyunsaturated fat, and the remaining 60 - 70% as monounsaturated fat and carbohydrate. The obese women have at least 1700 cal/day and at least 170 g of carbohydrates/day. Restricted carbohydrate intake may decrease fetal lean mass and increase body fat percent. The calorie requirement increases in women with a lower BMI to maintain adequate weight gain.
- Regular physical activity such as walking, and upper body exercises, seem to be safe and effective in improving insulin sensitivity. The usual recommendation is 30 minutes of exercise five or more days/per week.
- Taking prescribed medications.
- Periodic follow up schedule at antenatal care clinic.

**Part III:** Each diabetic pregnant woman was taught about precautionary practices against COVID-19 and fungal mycosis infections. It included the following practices:

- [Wear a mask in public when you interact with other people](https://www.cdc.gov/coronavirus/2019-ncov/prevent-getting-sick/about-face-coverings.html).
- [Keep a safe space between yourself and others](https://www.cdc.gov/coronavirus/2019-ncov/prevent-getting-sick/social-distancing.html) (stay at least 6 feet away, which is about 2 arm lengths).
- Avoid crowds and [poorly ventilated indoor spaces](https://www.cdc.gov/coronavirus/2019-ncov/prevent-getting-sick/Improving-Ventilation-Home.html).
- [Wash your hands](https://www.cdc.gov/handwashing/when-how-handwashing.html). If soap and water are not available, use a hand sanitizer with at least 60% alcohol.
- Avoid touching your eyes, nose, and mouth with unwashed hands.
- Cover coughs and sneezes with a tissue or the inside of your elbow. Then wash your hands.
- Clean frequently touched surfaces daily using household cleaners, such as soap or detergent.
- If **you are pregnant or were recently pregnant**[**you can receive a COVID-19 vaccine**](https://www.cdc.gov/coronavirus/2019-ncov/vaccines/recommendations/pregnancy.html)**, Keep all of your healthcare appointments during and after pregnancy.**
- Visit with your healthcare provider for all recommended appointments, Seek medical care immediately if you experience any [urgent maternal warning signs and symptoms](https://www.cdc.gov/hearher/maternal-warning-signs/index.html) (for example, a headache that won't go away, dizziness, fever, severe swelling of the hand, face, arm or leg, trouble breathing, chest pain or fast-beating heart, severe nausea and throwing up, or vaginal bleeding or discharge during or after pregnancy). These symptoms could indicate a potentially life-threatening complication.

1. **The Third Session**

It is concerned with diabetes self-care including insulin administration and blood glucose monitoring for good diabetic control.

- - 1. **Blood glucose measurement technique:**
- Blood sugar testing requires the use of a blood sugar meter. The meter monitors the amount of sugar in a small sample of blood, usually from your fingertip, that you place on a disposable test strip. The following steps were demonstrated practically to them :

1. Wash and dry hands well. (Food and other substances can give you an inaccurate reading.)
2. Insert a test strip into your meter.
3. Prick the side of your fingertip with the needle (lancet) provided with your test kit.
4. Touch and hold the edge of the test strip to the drop of blood.
5. The meter will display your blood sugar level on a screen after a few seconds.

- Some meters can test blood taken from an alternate site, such as the forearm or palm. But these readings may not be as accurate as readings from the fingertips, especially after a meal or during exercise, when blood sugar levels change more frequently.
  - 1. **Insulin administration technique**:

1. Wash hands with soap and water. This will help prevent infection. Dry hands with a clean towel or paper towel.
2. Clean the skin where you will inject the insulin. You can use an alcohol pad, or a cotton swab dipped in alcohol.
3. Grab a fold of skin. Gently pinch the skin and fat between the thumb and first finger.
4. Insert the needle straight into the skin. Do not hold the syringe at an angle. Make sure the needle is in the skin. Let go of the pinched tissue.
5. Push down on the plunger to inject the insulin. Press on the plunger until the insulin is gone. Keep the needle in place for 5 seconds after injecting the insulin.
6. Pull out the needle. Press on the injection site for 5 to 10 seconds. Do not rub. This will keep insulin from leaking out.
7. Throw away the used insulin syringe as directed. Do not recap the syringe before you throw it away.
8. **The Fourth session**

It included hygienic practices for the prevention of COVID -19 infection as follows:

1. **Hand washing technique in the right way:** Wet your hands with clean, running water (warm or cold), turn off the tap and apply soap. Lather your hands by rubbing them together with the soap. Lather the backs of your hands, between your fingers, and under your nails. Scrub your hands for at least 20 seconds. Rinse your hands well under clean, running water. Dry your hands using a clean towel or air-dry them**.**
2. **Respiratory protection is the wearing of cloth face covering and removal of cloth face covering safely**
3. **How to wear cloth face coverings (Mask) correctly***:* Wash your hands before putting on your face covering**.** Put it over your nose and mouth and secure it under your chin**.** Try to fit it snugly against the sides of your face**.** Make sure you can breathe easily.
4. **How to remove cloth face coverings (Mask) correctly:** Untie the strings behind your head or stretch the ear loops**.** Handle only by the ear loops or ties**.** Fold outside corners together**.** Be careful not to touch your eyes, nose, and mouth when removing, and wash hands immediately after removing.
